# Evaluation of clinical risk factors for developing pleural empyema secondary to liver abscess

**DOI:** 10.1186/s12876-019-1128-4

**Published:** 2019-12-16

**Authors:** Eunjue Yi, Tae Hyung Kim, Jun Hee Lee, Jae Ho Chung, Sungho Lee

**Affiliations:** 1 Department of Thoracic and Cardiovascular Surgery, Korea University College of Medicine, 73, Goryeodae-ro, Seongbuk-gu, Seoul, 02841 Republic of Korea; 2 Department of Internal Medicine, Korea University College of Medicine, 73, Goryeodae-ro, Seongbuk-gu, Seoul, 02841 Republic of Korea

**Keywords:** Liver abscess, Pleural empyema, Risk factors

## Abstract

**Background:**

The aim of this study was to investigate the clinical manifestation and predictive risk factors of pleural empyema developing during treatment of the pyogenic liver abscess.

**Methods:**

Medical records of patients with the liver abscess in our institution were reviewed retrospectively. Enrolled patients were classified into four groups; Group 1: patients without pleural effusion, Group 2: patients with pleural effusion and who were treated noninvasively, Group 3: patient with pleural effusion and who were treated with thoracentesis, and Group 4: patients with pleural effusion that developed into empyema. Patient characteristics, clinical manifestation, and possible risk factors in development of empyema were analyzed.

**Results:**

A total of 234 patients was enrolled in this study. The incidence rate of empyema was 4.27% (10 patients). The mean interval for developing pleural effusion was 5.6 ± 6.35 days. In multivariate analysis, risk factors for developing pleural effusion included the location of the liver abscess near the right diaphragm (segment 7 and 8, OR = 2.30, *p* = 0.048), and larger diameter of the liver abscess (OR = 1.02, *p* = 0.042). Among patients who developed pleural effusions, presences of mixed microorganisms from culture of liver aspirates (OR = 10.62, *p* = 0.044), bilateral pleural effusion (OR = 46.72, *p* = 0.012) and combined biliary tract inflammation (OR = 21.05, *p* = 0.040) were significantly associated with the need for invasive intervention including surgery on effusion.

**Conclusion:**

The location of the liver abscess as well as pleural effusion, elevated inflammatory markers, and combined biliary tract inflammation may be important markers of developing pleural complication in patients with pyogenic liver abscess.

## Background

The etiology of pleural effusion (PE) varies from benign inflammatory disease to malignancy [1]; however, empyema is largely a result of preceding pneumonia, thoracic surgery, or chest injury [2]. More than half of bacterial pneumonia cases are associated with parapneumonic PE [3]; the presence of alcoholism, leukocytosis (> 15,000mm^3^), or neutrophilia, (> 50% of counted leukocytes); or being male, all of which have been reported as predictive risk factors for empyema [4, 5]. Although it is uncommon, pyogenic liver abscess (PLA) substantially increases the risk of empyema by 18 times [6].

Pleural empyema following PLA is a rare but challenging condition that negatively impacts on the treatment process. Both diseases may require surgical intervention, with high risk of mortality and morbidity [2, 7]. In the past, pleural empyema combined with amoebic liver abscess has been reported sporadically. With the etiologic shift, *Klebsiella pneumoniae* is likely to occasionally be a causative agent [8].

Several studies have investigated the risk of metastatic infections following PLA, and showed that diabetes, alcoholism, and bacteremia could be independent risk factors for the metastatic infections [9]. *K. pneumonia* was associated with a higher incidence of extra-hepatic infections. However, there are few reports evaluating the risk of pleural empyema in association with PLA. Goumard et al. reported that his team had conducted the first study of pleural empyema followed by liver resection surgery [10]. In this study, right sided hepatic resection, intraabdominal sepsis along with postoperative bile leakage or history of diaphragm opening could be risk factors.

During the past decade, we have treated several empyema cases associated with PLA. Based on these cumulated experiences, we investigated and analyzed the possible risk factors of pleural empyema in patients with PLA, such as the presence and location of pleural effusion or microbiology. Early detection of empyema is important because optimal surgical intervention is essential for better treatment outcomes [11]. Predictive cautions could facilitate the therapeutic process of PLA with few sequelae.

## Methods

### Patient characteristics and initial clinical conditions

Medical records of patients who had been admitted and treated for PLA between October 2008 and December 2017 in our institution were reviewed retrospectively. This study has been approved by Institutional Review Board of Korea University Anam Hospital (IRB Number; 2019AN0183) and performed in accordance with the ethical guidelines of the 2008 Declaration of Helsinki. A waiver of informed consent was obtained.

Inclusion criteria were (1) patients older than 18, (2) patients diagnosed with PLA, and (3) patients who underwent percutaneous drainage and antibiotic administration. A total of 290 patients was candidates for this study; patients who had hepatocellular carcinoma or had received transarterial chemoembolization (TACE) for hepatocellular carcinoma (40 patients) and patients with cholangiocarcinoma (16 patients) were excluded.

Demographic characteristics were investigated through patient interview records. Smoking status was categorized into never (never smoked or smoked 100 or fewer cigarettes ever), former (smoked at least 100 cigarettes but had quit at the time of the interview), and current smoker (smoked at least 100 cigarettes and currently smoking) [12]. Amount of alcohol consumption was classified into none, mild (less than two drinks per week), moderate (more than two and less than five drinks per week), and excessive (five or more drinks per week). One drink was defined as consumption of 200 ml of a beverage with 15% alcohol content [13].

Presence of major comorbidities was described, and comorbidity scores were calculated according to the modified Charlson Comorbidity Index [14, 15]. Combined metastatic infectious conditions were identified according to the radiologic reports of chest and abdominal CT studies. The amount of ascites was estimated using abdominal CT and ultrasonographic examinations. The presence of combined biliary tract inflammation was defined as the presence of cholangitis, cholecystitis, choledocholithiasis, or other inflammatory condition observed in imaging studies at the time of PLA diagnosis.

The researchers also investigated the admission route of the patients, whether emergency (ED) or outpatient department. They also checked the units to which patients were first admitted—general ward or intensive care unit (ICU). The initial admission route and place could be important for indirectly estimating clinical severity. Demographic information and initial clinical data are described in Table 1.

**Table 1:** Demographic characteristics and initial clinical condition

Variables	Pt. PE (−) (*N* = 120)	Pt. PE (+) (*N* = 114)							Total (*N* = 234)	*P*-value
		Pt. with simple PE (*N* = 78)	Pt. with complicated PE (*N* = 36)				Total (*N* = 114)	*P*-value		
	Group1 (*N* = 120)	Group2 (*N* = 78)	Group3 (*N* = 26)	Group4 (*N* = 10)	Total (N = 36)	*P*-value				
Age	61.0 ± 14.1	63.6 ± 16.1	63.0 ± 14.0	60.2 ± 14.5	60.7 ± 12.54	0.892	63.1 ± 14.96	0.234	62.0 ± 14.54	0.627
Gender, male	78 (65.0)	51 (65.4)	17 (65.4)	4 (40.0)	21 (58.3)	0.260	72 (63.2)	0.533	150 (64.1)	0.299
Smoking						0.234		0.668		0.763
None	79 (65.8)	54 (62.9)	19 (73.1)	7 (70.0)	26 (72.2)		80 (70.2)		159 (67.9)	
Ex-smoker	16 (13.3)	11 (14.1)	1 (3.8)	2 (20.0)	3 (8.3)		14 (12.3)		30 (12.8)	
Current smoker	25 (20.8)	13 (16.7)	6 (23.1)	1 (10.0)	7 (19.4)		20 (17.5)		45 (19.2)	
Alcohol consumption (times/week)						0.712		0.335		0.618
None	73 (60.8)	41 (56.2)	15 (57.7)	6 (60.0)	21 (58.3)		65 (57.0)		138 (56.0)	
> 2	23 (19.2)	14 (19.2)	8 (30.8)	2 (20.0)	10 (27.8)		26 (22.8)		49 (20.9)	
2 ≤ and < 5	14 (11.7)	12 (16.4)	3 (11.5)	2 (20.0)	5 (13.9)		17 (14.9)		31 (13.2)	
5 ≤	10 (8.3)	6 (8.2)	0 (0.0)	0 (0.0)	0 (0.0)		6 (5.3)		16 (6.85)	
Underlying disease										
Diabetes mellitus	42 (35)	32 (41.0)	11 (42.3)	3 (30.0)	14 (38.9)	0.706	46 (40.4)	1.000	88 (37.6)	0.420
HbA1c	7.5 ± 2.5	7.6 ± 2.2	8.3 ± 2.8	6.6 ± 1.6	7.8 ± 2.5	0.645	7.7 ± 2.3	0.991	7.6 ± 2.4	0.876
Hypertension	52 (43.3)	38 (36.2)	10 (38.5)	5 (50.0)	15 (14.3)	0.469	53 (46.5)	0.547	105 (44.9)	0.694
Liver cirrhosis	3 (2.5)	2 (2.6)	1 (3.8)	0	1 (2.8)	1.000	3 (2.6)	1.000	6 (2.6)	1.000
HBV carrier	3 (2.5)	3 (3.8)	0	0	0	0.682	3 (2.5)	0.550	6 (2.6)	1.000
Chronic Kidney disease	4 (3.4)	6 (7.7)	2 (7.7)	2 (20.0)	4 (11.1)	0.198	10 (8.8)	0.723	14 (6.0)	0.101
Heart disease	1 (0.8)	7 (9.0)	4 (15.4)	1 (10.0)	5 (13.9)	1.000	12 (10.5)	0.514	13 (5.6)	0.001
Charlson comorbidity Index	2.5 ± 1.7	2.9 ± 1.8	2.9 ± 1.4	1.9 ± 1.5	2.7 ± 1.7	0.055	2.7 ± 1.7	0.236	2.7 ± 1.7	0.144
Combined inflammatory condition										
Biliary tract inflammation	23 (19.2)	25 (32.1)	6 (23.1)	6 (60.0)	12 (33.3)	0.053	37 (32.5)	1.000	60 (25.6)	0.025
Urinary tract infection	8 (6.7)	15 (19.2)	3 (11.5)	3 (30.0)	6 (16.7)	0.317	21 (18.4)	0.801	29 (12.4)	0.022
Acute kidney injury	6 (5.0)	11 (14.1)	1 (3.8)	3 (30.0)	4 (11.1)	0.057	15 (13.2)	0.500	21 (9.0)	0.038
Rupture of Abscess	2 (1.7)	2 (2.7)	0	2 (20.0)	2 (5.6)	0.071	4 (3.5)	0.590	6 (2.6)	0.437
Metastatic infection										
Sepsis	7 (5.8)	18 (23.1)	2 (7.7)	3 (30.0)	5 (13.9)	0.119	23 (20.2)	0.321	30 (12.8)	0.001
Pneumonia	4 (3.3)	5 (6.4)	0	0	0	–	5 (4.4)	0.178	9 (3.8)	0.744
Endophthalmitis	2 (1.7)	5 (6.4)	2 (7.7)	0	2 (5.6)	1.000	7 (6.1)	1.000	9 (3.8)	0.095
Septic pulmonary emboli	3 (2.5)	2 (2.6)	0	0	0	–	2 (1.8)	1.000	5 (2.1)	1.000
Peritonitis	5 (4.2)	6 (7.7)	1 (3.8)	1 (10.0)	2 (5.6)	0.484	8 (7.0)	1.000	13 (5.6)	0.401
Perirenal abscess	1 (0.8)	1 (1.3)	1 (3.8)	0 (0.0)	1 (2.8)	1.000	2 (1.8)	0.534	3 (1.3)	0.614
Psoas muscle abscess	1 (0.8)	1 (1.3)	0	1 (10.0)	1 (2.8)	0.278	2 (1.8)	0.534	3 (1.3)	0.614
Splenic abscess	1 (0.8)	2 (2.6)	0	1 (10.0)	1 (2.8)	0.278	3 (2.6)	1.000	4 (1.7)	0.359
Prostate abscess	2 (1.7)	4 (5.1)	3 (11.5)	0	3 (8.3)	0.545	7 (6.1)	0.677	9 (3.8)	0.095
Paravertebral abscess	1 (0.8)	1 (1.3)	0	0	0	–	1 (0.9)	1.000	2 (0.9)	1.000
Total	14 (11.7)	19 (24.4)	5 (19.2)	3 (30.0)	8 (23.7)	0.658	27 (23.7)	1.000	41 (17.5)	0.017
Combined ascites						0.320		0.513		< 0.000
None	102 (85.0)	46 (59.0)	15 (57.7)	5 (50.0)	20 (55.6)		66 (57.9)		168 (71.8)	
Small (< 500 cc)	15 (12.5)	21 (26.9)	9 (34.6)	4 (40.0)	13 (36.1)		34 (29.8)		49 (20.9)	
Moderate	1 (0.8)	10 (12.8)	2 (7.7)	0	2 (5.6)		12 (10.5)		13 (5.6)	
Large (> 1000 cc)	2 (1.7)	1 (1.3)	0	1 (10.0)	1 (2.8)		2 (1.8)		4 (1.7)	
Reactive lymph nodes	36 (30.0)	17 (21.8)	8 (30.8)	3 (30.0)	11 (30.6)	1.000	28 (24.6)	0.353	64 (27.4)	0.381
Admission route						0.689		0.500		0.014
Outpatients department	37 (30.8)	9 (11.5)	8 (30.8)	2 (20.0)	10 (27.8)		19 (16.7)		56 (23.9)	
Emergency department	83 (69.2)	69 (88.5)	18 (69.2)	8 (80.0)	26 (72.2)		95 (83.3)		178 (76.1)	
Admission ward						0.224		0.055		
General ward	113 (94.2)	55 (75.3)	20 (76.9)	5 (50.0)	25 (69.4)		84 (73.7)		197 (84.2)	< 0.001
Intensive care unit	7 (5.8)	18 (24.7)	6 (23.1)	5 (50.0)	11 (30.6)		30 (26.3)		37 (15.8)	

Data are presented as mean ± standard deviation and number (%). *Pt.* patients, *PE* pleural effusion, *HBV* hepatitis B virus

### Study group categorization and clinical manifestation

Detection of PE was dependent on the radiologic findings. The location of PE was described as the site at which PE first appeared—right, left, or bilateral. Intervals of PE were defined as the periods between diagnosis of PLA and detection of PE. The authors defined complicated PE as biochemical analyses that satisfied at least one of the following conditions: (1) pH < 7.20, (2) lactate dehydrogenase> 1000 IU/L, and (3) glucose< 60 mg/dL [3]. Pleural empyema was defined by performance of closed thoracostomy or video-assisted thoracoscopic surgery (VATS) drainage. When the PE disappeared spontaneously without any invasive procedures (thoracentesis for diagnostic and treatment purposes, chest tube insertion, and VATS drainage) during the treatment periods, we labeled this simple pleural effusion.

Patients included in this study were classified into PE (−), Group 1 and PE (+) groups. PE (+) groups were subdivided by treatment course into three groups. (1) Group 2: patients with simple and uncomplicated PE, (2) Group 3: patients with complicated PE who underwent thoracentesis, and (3) Group 4: patients with empyema. Clinical features of PLA, PE, laboratory findings, and treatment results of each group were investigated.

The location and numbers of PLA were identified using abdominal CT and ultrasonography. The locations were described in four ways, (1) segmental: one to eight segments, (2) sectional: left lateral, left medial, right anterior, and right posterior, (3) according to the relationship with the diaphragm (near right, near left, and non-related), and (4) according to lobe (right or left). The longest diameter of the PLA was measured. In cases of multiple PLA, the location and diameter of the largest were described. The microbiologic data of the PLA were based on culture reports performed on the pus obtained from percutaneous drainage. Microorganisms of the PLA were categorized into ***Klebsiella pneumoniae*** only, other single aerobic microorganisms such as *Escherichia coli* or *streptococcus*, mixed, and others including anaerobes.

### Statistical analysis

Clinical data were analyzed using IBM® SPSS® Statistics software Version 22.0 (IBM, Armonk, NY, USA). A chi-square test and Fisher’s exact test were used for analysis of categorical data. Student’s *t*-test and Mann-Whitney tests were used for continuous data.

Univariate analysis using a logistic regression model for identifying risk factors of PE between PE negative (Group 1) and PE positive (Groups 2, 3, and 4) patients was performed. Identical analysis was performed between Group 2 and patients with PE requiring intervention (thoracentesis, chest tube insertion, or surgery, Groups 3 and 4). Then, analysis was performed between Groups 3 and 4. Multivariate analysis was performed based on the results of univariate analysis. Statistical significance was defined as a *p* value less than 0.05.

## Results

### Demographic characteristics and initial clinical information

The study population was comprised of 234 patients. A total of 120 (51.3%) patients did not present with PE during the treatment period. Among the patients with PE (114 patients, 48.7%), 36 needed invasive intervention, and 10 (8.8% of PE positive patients) suffered from pleural empyema. Clinical manifestation of abscess, laboratory findings, and treatment results of each group are presented in Tables 2 and 3.

**Table 2:** Clinical manifestation of pyogenic liver abscess and pleural effusion

Variables	PE (−) (*N* = 120)	PE (+) (*N* = 114)							Total (*N* = 234)	*P*-value
	Group1 (*N* = 120)	Simple PE (*N* = 78)	Complicated PE (*N* = 36)				Total (*N* = 114)	*P*-value		
	Group1 (*N* = 120)	Group2 (*N* = 78)	Group3 (*N* = 26)	Group4 (*N* = 10)	Total (*N* = 36)	*P*-value				
Initial findings										
WBC, /mm3	13,366 ± 7016	14,261 ± 6794	14,267 ± 4447	16,232 ± 6126	13,887 ± 6663	0.293	14,436 ± 6255	0.479	13,887 ± 6663	0.103
Neutrophil, /mm3	11,164 ± 6082	12,483 ± 6322	15,045 ± 14,692	14,033 ± 4685	12,126 ± 7527	0.123	13,175 ± 8748	0.399	12,126 ± 7527	0.023
CRP, mg/dL	188 ± 79	206 ± 84	229 ± 71	286 ± 66	244 ± 73	0.462	219 ± 82	0.399	203 ± 82	0.007
First week follow-up										
WBC, /mm3	9254 ± 3410	11,109 ± 4549	12,610 ± 3770	19,506 ± 4558	14,526 ± 5500	0.001	12,188 ± 5100	0.001	10,696 ± 4558	< 0.001
Neutrophil, /mm3	6589 ± 3276	8680 ± 4298	10,019 ± 4083	16,477 ± 4468	11,826 ± 5598	0.003	9725 ± 4960	0.003	8137 ± 4468	< 0.001
CRP, mg/dL	54 ± 54	89 ± 67	98 ± 63	173 ± 66	76 ± 66	0.007	98 ± 70	0.036	76 ± 66	< 0.001
Second week follow-up										
WBC, /mm3	6484 ± 2111	7006 ± 2282	8011 ± 2270	10,332 ± 2607	8656 ± 2557	0.012	7541 ± 2487	0.001	7048 ± 2373	0.003
Neutrophil, /mm3	3816 ± 1816	4699 ± 2169	5340 ± 1956	7780 ± 2101	6037 ± 2263	0.003	5125 ± 2277	0.007	4512 ± 2170	< 0.001
CRP, mg/dL	11 ± 17	29 ± 32	35 ± 39	76 ± 47	47 ± 45	0.009	35 ± 38	0.039	24 ± 32	< 0.001
Location of largest abscess										
Segmental location						0.343		0.135		0.048
1	4 (3.3)	1 (1.3)	0	0	0		1 (0.9)		5 (2.1)	
2	8 (6.7)	6 (7.7)	3 (11.5)	2 (20.0)	5 (13.9)		11 (9.6)		19 (8.1)	
3	11 (9.2)	3 (3.8)	1 (3.8)	0	1 (2.8)		4 (3.5)		15 (6.4)	
4	19 (15.8)	14 (17.9)	3 (11.5)	0	3 (8.3)		17 (14.9)		36 (15.4)	
5	14 (11.7)	3 (3.8)	2 (7.7)	2 (20.0)	4 (11.1)		7 (6.1)		21 (9.0)	
6	23 (19.2)	12 (15.4)	3 (11.5)	1 (10.0)	4 (11.1)		16 (14.0)		39 (16.7)	
7	15 (12.5)	16 (20.5)	12 (46.2)	2 (20.0)	14 (38.9)		30 (26.3)		45 (19.2)	
8	26 (21.7)	23 (29.5)	2 (7.7)	3 (30.0)	5 (13.9)		28 (24.6)		54 (23.1)	
Association with diaphragm						0.910		0.924		0.007
lower parts (1, 3, 5, 6)	52 (43.3)	19 (24.4)	6 (23.1)	3 (30.0)	9 (25.0)		28 (24.6)		80 (34.2)	
Near Lt. diaphragm (2, 4)	27 (22.5)	20 (25.6)	6 (23.1)	2 (20.0)	8 (22.2)		28 (24.6)		55 (23.5)	
Near Rt. diaphragm (7, 8)	41 (34.2)	39 (50.0)	14 (53.8)	5 (50.0)	19 (52.8)		58 (50.9)		99 (42.3)	
Sectional locations						0.303		0.167		0.500
Left lateral section (2, 3)	19 (15.8)	9 (11.5)	4 (15.4)	2 (20.0)	6 (16.7)		15 (13.2)		34 (14.5)	
Left medial section (1, 4)	23 (19.2)	15 (19.2)	3 (11.5)	0	3 (8.3)		18 (15.8)		41 (17.5)	
Right anterior section (8, 5)	40 (33.3)	26 (33.3)	4 (15.4)	4 (40.0)	8 (22.2)		34 (29.8)		74 (31.6)	
Right posterior section (6, 7)	38 (31.7)	28 (35.9)	15 (57.7)	4 (40.0)	19 (52.8)		47 (41.2)		85 (36.3)	
Lobes						1.000		0.658		0.331
Left lobe	42 (35.0)	24 (30.8)	7 (26.9)	2 (20.0)	9 (25.0)		33 (28.9)		75 (32.1)	
Right lobe	78 (65.0)	54 (69.2)	19 (73.1)	8 (80.0)	27 (75.0)		81 (71.1)		159 (67.9)	
Largest diameter of abscess (mm)	56.8 ± 23.15	62.1 ± 26.40	69.9 ± 27.42	75.0 ± 25.55	71.3 ± 26.65	0.715	65.0 ± 26.71	0.073	60.8 ± 25.24	0.019
Number of abscess						0.585		0.768		0.089
1	93 (77.5)	58 (74.4)	20 (76.9)	9 (90.0)	29 (80.6)		87 (76.3)		180 (76.9)	
2	16 (13.3)	6 (7.7)	2 (7.7)	0	2 (5.6)		8 (7.0)		24 (10.3)	
≥ 3	11 (9.2)	14 (17.9)	4 (15.4)	1 (10.0)	5 (13.9)		19 (16.7)		30 (12.8)	
Culture results of abscess						0.096		0.213		0.565
None	25 (20.8)	18 (23.1)	4 (15.4)	1 (10.0)	5 (13.9)		23 (20.2)		48 (20.5)	
*K. pneumoniae*	72 (60.0)	42 (53.8)	16 (61.5)	3 (30.0)	19 (52.8)		61 (53.5)		133 (56.8)	
Other gram (+)	17 (14.2)	16 (20.5)	5 (19.2)	3 (30.0)	8 (22.2)		24 (21.1)		41 (17.5)	
Mixed	6 (5.0)	2 (2.6)	1 (3.8)	3 (30.0)	4 (11.1)		6 (5.3)		12 (5.1)	
Blood culture results						0.118		0.863		0.121
None	81 (67.5)	41 (52.6)	17 (65.4)	5 (50.0)	22 (61.1)		63 (55.3)		144 (61.5)	
K. pneumoniae	27 (22.5)	29 (37.2)	8 (30.8)	3 (30.0)	11 (30.6)		40 (35.1)		67 (28.6)	
Other gram (+)	10 (8.3)	5 (6.4)	0	2 (20.0)	2 (5.6)		7 (6.1)		17 (7.3)	
Mixed	2 (1.7)	3 (3.8)	1 (3.8)	0	1 (2.8)		4 (3.5)		6 (2.6)	
Recurrence of PLA	4(3.3)	3 (3.8)	3 (11.5)	2 (20.0)	5 (13.9)	0.603	8 (7.0)	0.107	12 (5.1)	0.244
Location of PE						0.006		0.919		
Left		3 (3.8)	1 (3.8)	1 (10.0)	5 (5.6)		5 (4.4)			
Right		42 (53.8)	18 (69.2)	1 (10.0)	19 (52.8)		61 (53.5)			
Bilateral		33 (42.3)	7 (26.9)	8 (80.0)	15 (41.7)		48 (42.1)			
Presence of PE at diagnosis		26 (33.3)	10 (38.5)	5 (50.0)	15 (41.7)	0.709	41 (36.0)	0.408		
First presence of PE (day)		5.5 ± 5.61	6.0 ± 8.49	5.3 ± 6.1	5.8 ± 7.82	0.821	5.6 ± 6.35	0.857		
PAD duration	16.5 ± 34.45	18.4 ± 13.33	21.1 ± 8.52	28.1 ± 5.20	23.0 ± 8.30	0.014	19.9 ± 12.13	0.001	18.1 ± 26.08	< 0.001

Data are presented as mean ± standard deviation and number (%). Normal ranges of variables are presented as follows: WBC, 4000–10,000 /mm3; Neutrophil, 38–75% of WBC; CRP, 0–5.0 mg/dL. *PE* pleural effusion, *WBC* white blood cell, *CRP* C-reactive protein, *PLA* pyogenic liver abscess, *PAD* percutaneous abscess drainage

**Table 3:** Treatment results of each groups

Variables	PE (−) (*N* = 120)	PE (+) (*N* = 114)							Total (*N* = 234)	*P*-value
	Group1 (*N* = 120)	Pt. with Simple PE (*N* = 78)	Pt. with complicated pleural effusion (*N* = 36)				Total (*N* = 114)	*P*-value		
	Group1 (*N* = 120)	Group2 (*N* = 78)	Group3 (*N* = 26)	Group4 (*N* = 10)	Total (*N* = 36)	*P*-value				
Results										
PAD duration	16.5 ± 34.5	18.4 ± 13.3	21.1 ± 8.5	28.1 ± 5.2	23.0 ± 8.3	0.014	19.9 ± 12.1	0.001	18.1 ± 26.1	< 0.001
Hospital stays, days	21.2 ± 29.4	24.1 ± 13.2	24.0 ± 8.6	38.5 ± 12.7	28.1 ± 11.7	< 0.000	25.4 ± 12.9	0.128	23.2 ± 22.9	0.163
Treatment periods, days	45.9 ± 21.6	50.6 ± 23.6	54.3 ± 24.3	91.5 ± 47.8	64.6 ± 36.0	0.009	55.0 ± 28.7	0.022	50.4 ± 25.7	0.018
Discharge without complication	112 (93.3)	71 (91.0)	22 (84.6)	6 (60.0)	28 (77.8)	0.179	99 (86.8)	0.073	211 (90.0)	0.124
Recurrence	4 (3.3)	3 (3.8)	3 (11.5)	2 (20.0)	5 (13.9)	0.603	8 (7.0)	0.107	12 (5.1)	0.244
Readmission due to complication, ≤90 days	3 (2.5)	2 (2.6)	2 (7.7)	0	2 (5.6)	1.000	4 (3.5)	0.590	7 (3.0)	0.716
Hopeless discharge	0	3 (3.8)	1 (3.8)	0	1 (2.8)	1.000	4 (3.5)	1.000	4 (1.7)	0.055
Death	3 (2.5)	2 (2.6)	0	0	0	–	2 (1.8)	1.000	5 (2.1)	1.000

PAD duration and treatment periods of each group showed statistically significant differences. Hospital stay Group 4 (Empyema) was significantly longer than those of group 3, however, those of Group 2 and patients with complicated PE (Group 3 and 4) showed no differences, neither between Group 1 and other groups. Discharge rates without complication, recurrence rates, readmission rate, and mortality rates showed no significant differences between each group. Data are presented as mean ± standard deviation and number (%). *Pt.* patients, *PE* pleural effusion, *PAD* percutaneous abscess drainage

### Risk factors of pleural effusion and need for interventional methods including surgery

The mean interval of developing pleural effusion was 5.6 ± 6.35 days. Multivariate analysis demonstrated that elevated CRP level at the second week post-PLA diagnosis (*p* = 0.001), location near the right diaphragm (segments 7 and 8, *p* = 0.048), and larger liver abscess diameter (*p* = 0.042) were statistically significant risk factors of pleural effusion (Table 4).

**Table 4:** Multivariate analysis for investigating risk factors of developing PE, complicated PE and pleural empyema

Variables	OR	95% Confidential Interval	*P*-value
Multivariate analysis for risk factors of PE			
Heart Disease	2.88	0.000–0.000	0.998
Admission via ER	1.69	0.688–4.149	0.252
Admission to ICU	1.98	0.568–6.900	0.284
Biliary tract inflammation	1.78	0.757–4.198	0.186
Urinary tract infection	1.70	0.560–5.150	0.349
Acute kidney injury	1.85	0.434–7.863	0.406
Sepsis	2.60	0.688–9.851	0.159
Total metastatic infections	0.64	0.213–1.894	0.416
Combined ascites			0.352
small	1.42	0.573–3.541	0.447
moderate	6.80	0.705–65.598	0.097
large	0.79	0.060–10.519	0.860
Second week follow-up CRP, mg/dL	1.04	1.016–1.061	0.001
Location associated with diaphragm			0.137
Near left diaphragm (2, 4)	1.72	0.694–4.259	0.242
Near right diaphragm (7, 8)	2.30	1.008–5.265	0.048
Largest diameter of abscess (mm)	1.02	1.001–1.034	0.042
Multivariate analysis for risk factors of complicated PE			
Admission via ER	0.41	0.120–1.378	0.148
Initial CRP, mg/dL	1.01	0.999–1.013	0.090
1st week follow-up CRP, mg/dL	1.00	0.990–1.008	0.830
2nd week follow-up CRP, mg/dL	1.01	0.993–1.030	0.236
Culture results of abscess			
K. pneumoniae	1.35	0.296–6.170	0.697
Other gram(+)	2.70	0.490–14.823	0.254
Mixed	10.62	1.069–105.411	0.044
Multivariate analysis for risk factors of pleural empyema			
Biliary tract inflammation	21.05	1.152–384.785	0.040
Urinary tract infection	3.07	0.202–46.678	0.419
Location of PE			
Right	12.63	0.249–640.051	0.205
Bilateral	46.72	2.354–927.452	0.012
Association with diaphragm			0.642
Near left diaphragm (2, 4)	2.79	0.115–67.482	0.528
Near right diaphragm (7, 8)	0.50	0.047–5.348	0.567

*PE* pleural effusion, *OR* odds ratio, *ER* emergency room, *ICU* intensive care unit, *CRP* C-reactive protein. Normal ranges of variables are presented as follows: WBC, 4000–10,000 /mm3; Neutrophil, 38–75% of WBC; CRP, 0–5.0 mg/dL

Risk factors for interventions including thoracentesis, chest tube insertion, and surgery comprised microbial culture results from pus drained from the PLA. When the isolated organisms were mixed with several types of gram positive and negative species, the hazard ratio of need for intervention increased to 10.62 (*p* = 0.044).

Pleural empyema developed in 10 patients (4.3% in total patients, 8.8% in PE positive patients, and 27.8% in invasively treated patients). Five patients were cured by chest tube insertion, and five patients required surgery (three patients underwent initial chest tube drainage, and two patients underwent initial VATS drainage without chest tube insertion). Risk factors of empyema in patients who needed interventions were presence of combined biliary tract inflammation (*p* = 0.004) and bilateral pleural effusion (*p* = 0.012). The results of univariate and multivariate analysis are summarized in Additional file 1: Table S1 and Table 4.

## Discussion

Out of 234 PLA patients in the past decade, we noted 10 cases of pleural empyema, with five cases needing surgical decortication. The estimated incidence rate was very low, only 4.3%, and it seemed difficult to evaluate predictive risk factors directly through multivariate analysis. Therefore, we started by investigating risk factors of PE to identify the possible conditions that contribute to pleural empyema.

When the enrolled patients were divided into PE (−) and PE (+) groups, multivariate analysis revealed three statistically significant risk factors: (1) location of PLA near the right diaphragm (segments 7 and 8), (2) larger abscess size, and (3) elevated CRP level at the second week post- diagnosis. However, considering that the median interval from diagnosis of PLA to appearance of PE was 5.6 days (±6.35, ranging 4.4 to 6.7), the increased numbers might be explained as results rather than causes. As the estimated hazard ratio of larger abscess size was 1.02, this may have little effect on PE.

Patients with PLA located near the right diaphragm were at higher risk—more than double—of developing pleural effusion. Hydrothorax-associated hepatic diseases have been reported as largely dependent of right sides, because the ascites moves through diaphragmatic defects along pressure gradients [16]. The occurrence of pleural empyema after liver resection is also known to be primarily related to right hepatectomy [11]. There were reports that upper abdominal surgery induced significant postoperative changes in the surface electromyogram in the diaphragm [17]. Thus, irritation due to surgery or inflammation near the right diaphragm is a possible cause of PE.

Although PE had appeared, it will not harmful if it does not developed into empyema. In our study, 31.6% of simple PE progressed to complicated cases (36 of 114 patients), and multivariate analysis revealed that presence of mixed gram (+) and gram (−) microorganisms in the culture of pus drained from the liver abscess was a significant risk factor (HR = 10.62). *K. pneumoniae*, which is the most common etiology of PLA [6] and is frequently complicated by metastatic infections such as bacteremia, sepsis, endophthalmitis, pulmonary infection, or intraabdominal abscess [8], was not associated with increased risk for complicated PE (*p* = 0.697). Complicated PE is often related to pneumonia [3], which can manifest as a metastatic infection in 12.6% of PLA patients [6]. However, presence of pneumonia was not significantly associated with development of complicated PE in our study (*p* = 0.178).

This is likely to suggest that changes from simple to complicated PE are due to failure of antibiotic treatment, because the initial treatment choice of PLA is percutaneous drainage and third-generation cephalosporin with or without metronidazole [7]. Empirical antibiotic application could have effects on mainstream microorganisms but not for unusual and mixed species (Regimens and change rates of antibiotics in our study were described in Additional file 2: Table S2). No microorganisms were cultured from drained pleural effusion, including empyema; therefore, development of complicated PE might not be due to metastatic infection.

The etiology of PLA did not seem to influence development of empyema from complicated PE. Multivariate analysis revealed that combined biliary tract inflammation (choledocholithiasis, cholangitis, or cholecystitis) and presence of bilateral PE was significantly associated with pleural empyema (Univariate and multivariate analysis of risk factors for bilateral PE were described in Additional file 2: Table S3). Combined biliary tract inflammation has been reported as a common causative condition for PLA [7, 18]. A total of 60 patients (25.6%) had combined biliary tract inflammation (cholangitis in 9, cholecystitis in 48, choledocholithiasis in six, one GB perforation and one cholangiohepatitis), 36 in PE (+) patients (25.2%), 12 in complicated PE (33.3%), and six in empyema cases (60%) in the current study (Additional file 2: Table S4). The proportion of combined biliary tract inflammation was increased in empyema patients: this might indicate that obstruction of the biliary tract could deteriorate the liver abscess and then interfere with antibiotics and increased risks for empyema. The presence of bilateral PE might reflect relatively extensive effusion, which is seldom resolved spontaneously or treated by thoracentesis, and therefore could be aggravated.

This study had several limitations. First, because the study population was small, we had to evaluate risk factors indirectly. Second, we did not fully investigate antibiotic therapies due to the complexity of various combinations during the treatment period. For more accurate assessment of antibiotic effects, further examination is needed regarding antibiotic combinations and changes. Finally, more detailed analysis for initial inflammatory conditions in PLA patients, including laboratory tests, should be implemented. Because there were few patients who underwent regular laboratory test follow-up for identifying inflammatory conditions, including procalcitonin, we could not perform more precise analysis on laboratory findings. Initial and follow-up changes in inflammatory markers are important factors to tracing successful application of treatment strategies.

## Conclusions

The location of liver abscess near the right diaphragm, detection of mixed microorganisms in pus drained from liver abscess, and combined biliary tract disease could affect the development of pleural complications requiring invasive procedures such as chest tube insertion and surgical debridement. In multivariate analysis, combined biliary tract inflammation and bilateral pleural effusion were identified as significant risk factors for pleural empyema.

## Supplementary information

**Additional file 1: Table S1.** Univariate analysis for investigating risk factors of developing pleural effusion, complicated pleural effusion and empyema.

**Additional file 2: Table S2.** Antibiotics treatment regimen. **Table S3.** Univariate and Multivariate analysis of risk factors for bilateral pleural effusion. **Table S4.** Classification of combined biliary tract disease according to the Groups.

## Data Availability

The datasets generated and/or analyzed during the current study are not publicly available due personal medical records, but are available from the corresponding author on reasonable request and approval of IRB.

## Abbreviations

ICU: Intensive care unit
OR: Odds ratio
PE: Pleural effusion
PLA: Pyogenic liver abscess
TACE: Transarterial chemoembolization
VATs: Video-assisted thoracoscopic surgery
