# Supplementary material for: Evaluation of clinical risk factors for developing pleural empyema secondary to liver abscess
Source: BMC Gastroenterol. 2019 Dec 16;19:215. doi: 10.1186/s12876-019-1128-4 (PMC6915871; doi:10.1186/s12876-019-1128-4)
Supplement: Supplementary file 1 — Additional file 1: Table S1. Univariate analysis for investigating risk factors of developing pleural effusion, complicated pleural effusion and empyema. [file 12876_2019_1128_MOESM1_ESM.docx]

**Additional file 1:** Table S1 Univariate analysis for investigating risk factors of developing pleural effusion, complicated pleural effusion and empyema.

| **Variables** | | | **Pleural effusion** | | | | | **Intervention** | | | | | **Pleural empyema** | | | | |
| --- | --- | --- | --- | --- | --- | --- | --- | --- | --- | --- | --- | --- | --- | --- | --- | --- | --- |
|  | | | **OR** | **95% Confidential Interval** | | | ***P*-value** | **OR** | **95% Confidential Interval** | | | ***P*-value** | **OR** | **95% Confidential Interval** | | | ***P*-value** |
|  | Age at diagnosis | | 1.01 | 0.992 | – | 1.029 | 0.275 | 0.98 | 0.959 | – | 1.010 | 0.233 | 1.00 | 0.939 | – | 1.056 | 0.888 |
|  | Men | | 0.99 | 0.572 | – | 1.723 | 0.980 | 0.74 | 0.330 | – | 1.667 | 0.469 | 0.35 | 0.079 | – | 1.584 | 0.174 |
|  | Smoking history | |  |  |  |  |  |  |  |  |  |  |  |  |  |  | 0.299 |
|  |  | Ex-smoker | 0.76 | 0.330 | – | 1.750 | 0.519 | 0.57 | 0.145 | – | 2.206 | 0.413 | 5.43 | 0.423 | – | 69.670 | 0.194 |
|  |  | Current | 0.68 | 0.336 | – | 1.375 | 0.283 | 1.12 | 0.399 | – | 3.136 | 0.832 | 0.45 | 0.046 | – | 4.456 | 0.497 |
|  | Alcohol consumption (times/week) | |  |  |  |  |  |  |  |  |  |  |  |  |  |  | 0.718 |
|  |  | < 2 | 1.39 | 0.709 | – | 2.740 | 0.335 | 1.31 | 0.509 | – | 3.372 | 0.576 | 0.63 | 0.102 | – | 3.841 | 0.612 |
|  |  | 2≤, and < 5 | 1.60 | 0.690 | – | 3.730 | 0.272 | 0.87 | 0.272 | – | 2.801 | 0.819 | 1.67 | 0.220 | – | 12.617 | 0.621 |
|  |  | 5 ≤ | 0.71 | 0.241 | – | 2.082 | 0.530 | 0.00 | 0.000 | – |  | 0.999 |  |  |  |  |  |
|  | Comorbidity | |  |  |  |  |  |  |  |  |  |  |  |  |  |  |  |
|  |  | Diabetes mellitus | 1.14 | 0.644 | – | 2.010 | 0.657 | 0.91 | 0.408 | – | 2.052 | 0.829 | 0.58 | 0.123 | – | 2.782 | 0.500 |
|  |  | HbA1c | 1.03 | 0.895 |  | 1.185 | 0.679 | 1.02 | 0.821 | – | 1.262 | 0.874 | 0.67 | 0.382 | – | 1.168 | 0.157 |
|  |  | Hypertension | 0.93 | 0.534 | – | 1.614 | 0.793 | 0.75 | 0.339 | – | 1.669 | 0.483 | 1.60 | 0.368 | – | 6.959 | 0.531 |
|  |  | Liver cirrhosis | 1.06 | 0.203 | – | 5.508 | 0.948 | 1.09 | 0.095 | – | 12.377 | 0.947 | 0.00 | 0.000 | – |  | 1.000 |
|  |  | HBV carrier | 1.20 | 0.235 | – | 6.084 | 0.830 | 0.00 | 0.000 | – |  | 0.999 |  |  |  |  |  |
|  |  | Chronic kidney disease | 2.16 | 0.618 | – | 7.572 | 0.227 | 1.50 | 0.369 | – | 5.682 | 0.551 | 3.00 | 0.361 | – | 24.919 | 0.309 |
|  |  | Heart disease | 12.65 | 1.589 | – | 100.692 | 0.016 | 1.64 | 0.482 | – | 5.556 | 0.430 | 0.61 | 0.060 | – | 6.246 | 0.678 |
|  | Charlson comorbidity index | | 1.10 | 0.945 | – | 1.285 | 0.215 | 0.86 | 0.678 | – | 1.100 | 0.235 | 0.66 | 0.390 | – | 1.123 | 0.126 |
|  | Combined inflammatory condition | |  |  |  |  |  |  |  |  |  |  |  |  |  |  |  |
|  |  | Biliary tract inflammation | 2.03 | 1.112 | – | 3.694 | 0.021 | 1.06 | 0.457 | – | 2.456 | 0.892 | 5.00 | 1.051 | – | 23.789 | 0.043 |
|  |  | Abscess rupture | 2.15 | 0.385 | – | 11.947 | 0.384 | 2.24 | 0.302 | – | 16.537 | 0.431 | 1.00 | 0.000 | – | 0.000 | 0.999 |
|  |  | Urinary tract infection | 3.16 | 1.338 | – | 7.467 | 0.009 | 0.84 | 0.296 | – | 2.381 | 0.743 | 3.29 | 0.538 | – | 20.081 | 0.198 |
|  |  | Acute kidney injury | 2.88 | 1.076 | – | 7.703 | 0.035 | 0.76 | 0.225 | – | 2.577 | 0.661 | 10.71 | 0.959 | – | 119.696 | 0.054 |
|  | Metastatic infection | |  |  |  |  |  |  |  |  |  |  |  |  |  |  |  |
|  |  | Sepsis | 4.08 | 1.676 | – | 9.935 | 0.002 | 0.54 | 0.182 | – | 1.586 | 0.261 | 5.14 | 0.712 | – | 37.151 | 0.150 |
|  |  | Pneumonia | 1.33 | 0.348 | – | 5.083 | 0.676 | 0.00 | 0.000 | – |  | 0.999 |  |  |  |  |  |
|  |  | Endophthalmitis | 3.86 | 0.785 | – | 18.986 | 0.097 | 0.86 | 0.159 | – | 4.652 | 0.860 | 0.00 | 0.000 | – | 0.000 | 0.999 |
|  |  | Septic pulmonary emboli | 0.70 | 0.114 | – | 4.246 | 0.695 | 0.00 | 0.000 | – |  | 0.999 |  |  |  |  |  |
|  |  | Peritonitis | 1.74 | 0.551 | – | 5.472 | 0.346 | 0.71 | 0.135 | – | 3.681 | 0.679 | 2.78 | 0.157 | – | 49.218 | 0.468 |
|  |  | Perirenal abscess | 2.12 | 0.190 | – | 23.761 | 0.541 | 2.20 | 0.134 | – | 36.195 | 0.581 | 0.00 | 0.000 | – | 0.000 | 1.000 |
|  |  | Psoas muscle abscess | 2.12 | 0.190 | – | 23.761 | 0.541 | 2.20 | 0.134 | – | 36.195 | 0.581 | 1.00 | 0.000 | – | 0.000 | 1.000 |
|  |  | Splenic abscess | 3.22 | 0.330 | – | 31.378 | 0.315 | 1.09 | 0.095 | – | 12.377 | 0.947 | 1.00 | 0.000 | – | 0.000 | 1.000 |
|  |  | Prostate abscess | 3.86 | 0.785 | – | 18.986 | 0.097 | 1.68 | 0.356 | – | 7.941 | 0.512 | 0.00 | 0.000 | – | 0.000 | 0.999 |
|  |  | Paravertebral abscess | 1.05 | 0.065 | – | 17.038 | 0.971 | 0.00 | 0.000 | – |  | 1.000 |  |  |  |  |  |
|  |  | Total | 2.35 | 1.161 |  | 4.756 | 0.018 | 0.89 | 0.346 | – | 2.273 | 0.803 | 1.80 | 0.340 | – | 9.538 | 0.490 |
|  | Combined ascites | |  |  |  |  |  |  |  |  |  |  |  |  |  |  |  |
|  |  | small | 3.50 | 1.771 | – | 6.927 | 0.000 | 1.42 | 0.598 | – | 3.393 | 0.425 | 1.33 | 0.282 | – | 6.300 | 0.717 |
|  |  | moderate | 18.55 | 2.356 | – | 145.996 | 0.006 | 0.46 | 0.092 | – | 2.293 | 0.343 | 0.00 | 0.000 | – | 0.000 | 0.999 |
|  |  | large | 1.55 | 0.212 | – | 11.241 | 0.667 | 2.30 | 0.137 | – | 38.629 | 0.563 | 1.00 | 0.000 | – | 0.000 | 1.000 |
|  | Reactive Lymph nodes | | 0.76 | 0.426 | – | 1.354 | 0.352 | 1.58 | 0.648 | – | 3.844 | 0.314 | 0.96 | 0.197 | – | 4.721 | 0.964 |
|  | Admission via ER | | 2.23 | 1.191 | – | 4.171 | 0.012 | 0.34 | 0.124 | – | 0.928 | 0.035 | 1.78 | 0.306 | – | 10.324 | 0.521 |
|  | Admission to ICU | | 5.77 | 2.416 | – | 13.759 | 0.000 | 1.37 | 0.568 | – | 3.286 | 0.486 | 3.33 | 0.715 | – | 15.535 | 0.125 |
|  | ICU care during treatment periods | | 5.15 | 2.481 | – | 10.702 | 0.000 | 0.94 | 0.410 | – | 2.177 | 0.893 | 5.00 | 1.051 | – | 23.789 | 0.043 |
|  | Use of Mechanical ventilation | | 5.46 | 0.628 | – | 47.461 | 0.124 | 0.53 | 0.057 | – | 4.905 | 0.575 | 1.00 | 0.000 | – | 0.000 | 1.000 |
|  | Laboratory findings | |  |  |  |  |  |  |  |  |  |  |  |  |  |  |  |
|  | Initial findings | |  |  |  |  |  |  |  |  |  |  |  |  |  |  |  |
|  |  | WBC | 1.00 | 1.000 | – | 1.000 | 0.224 | 1.00 | 1.000 | – | 1.000 | 0.661 | 1.00 | 1.000 | – | 1.000 | 0.288 |
|  |  | Neutrophils | 1.00 | 1.000 | – | 1.000 | 0.290 | 1.00 | 1.000 | – | 1.000 | 0.248 | 1.00 | 1.000 | – | 1.000 | 0.837 |
|  |  | CRP | 1.00 | 1.001 | – | 1.008 | 0.021 | 1.01 | 1.001 | – | 1.011 | 0.029 | 1.01 | 0.999 | – | 1.026 | 0.060 |
|  | First week follow-up | |  |  |  |  |  |  |  |  |  |  |  |  |  |  |  |
|  |  | WBC | 1.00 | 1.000 | – | 1.000 | 0.000 | 1.00 | 1.000 | – | 1.000 | 0.002 | 1.00 | 1.000 | – | 1.001 | 0.007 |
|  |  | Neutrophils | 1.00 | 1.000 | – | 1.000 | 0.000 | 1.00 | 1.000 | – | 1.000 | 0.004 | 1.00 | 1.000 | – | 1.000 | 0.010 |
|  |  | CRP | 1.00 | 1.001 | – | 1.008 | 0.006 | 1.01 | 1.000 | – | 1.012 | 0.050 | 1.02 | 1.003 | – | 1.036 | 0.017 |
|  | Second week follow-up | |  |  |  |  |  |  |  |  |  |  |  |  |  |  |  |
|  |  | WBC | 1.00 | 1.000 | – | 1.000 | 0.002 | 1.00 | 1.000 | – | 1.000 | 0.002 | 1.00 | 1.000 | – | 1.001 | 0.022 |
|  |  | Neutrophils | 1.00 | 1.000 | – | 1.000 | 0.000 | 1.00 | 1.000 | – | 1.000 | 0.006 | 1.00 | 1.000 | – | 1.001 | 0.010 |
|  |  | CRP | 1.04 | 1.024 | – | 1.064 | 0.001 | 1.01 | 1.001 | – | 1.024 | 0.027 | 1.04 | 1.017 | – | 1.059 | 0.000 |
|  | Location of largest abscess | |  |  |  |  |  |  |  |  |  |  |  |  |  |  |  |
|  | Segmental location | |  |  |  |  |  |  |  |  |  |  |  |  |  |  |  |
|  |  | Segment 2 | 5.50 | 0.513 | – | 59.014 | 0.159 | 1.00 | 0.000 | – | 0.000 | 1.000 | 0.00 | 0.000 | – | 0.000 | 1.000 |
|  |  | Segment 3 | 1.45 | 0.123 | – | 17.233 | 0.766 | 1.00 | 0.000 | – | 0.000 | 1.000 | 0.00 | 0.000 | – | 0.000 | 0.999 |
|  |  | Segment 4 | 3.58 | 0.364 | – | 35.233 | 0.274 | 1.00 | 0.000 | – | 0.000 | 1.000 | 1.50 | 0.106 | – | 21.312 | 0.765 |
|  |  | Segment 5 | 2.00 | 0.187 | – | 21.431 | 0.567 | 1.00 | 0.000 | – | 0.000 | 1.000 | 0.50 | 0.028 | – | 8.952 | 0.638 |
|  |  | Segment 6 | 2.78 | 0.284 | – | 27.268 | 0.379 | 1.00 | 0.000 | – | 0.000 | 1.000 | 0.25 | 0.024 | – | 2.577 | 0.244 |
|  |  | Segment 7 | 8.00 | 0.821 | – | 78.000 | 0.074 | 1.00 | 0.000 | – | 0.000 | 1.000 | 2.25 | 0.179 | – | 28.254 | 0.530 |
|  |  | Segment 8 | 4.31 | 0.452 | – | 41.091 | 0.204 | 1.00 | 0.000 | – | 0.000 | 1.000 |  |  |  |  |  |
|  | Association with diaphragm | |  |  |  |  |  |  |  |  |  |  |  |  |  |  |  |
|  |  | Near left diaphragm (2, 4) | 1.93 | 0.956 | – | 3.880 | 0.067 | 0.84 | 0.270 | – | 2.642 | 0.771 | 2.03 | 0.928 |  | 4.428 | 0.076 |
|  |  | Near right. diaphragm (7, 8) | 2.63 | 1.429 | – | 4.831 | 0.002 | 1.03 | 0.392 | – | 2.697 | 0.954 | 2.60 | 1.313 |  | 5.161 | 0.006 |
|  | Sectional location | |  |  |  |  |  |  |  |  |  |  |  |  |  |  |  |
|  |  | Left medial section (1, 4) | 0.99 | 0.397 | – | 2.477 | 0.985 | 0.30 | 0.060 | – | 1.506 | 0.144 | 0.00 | 0.000 |  |  | 0.999 |
|  |  | Right. anterior section (8, 5) | 1.08 | 0.476 | – | 2.437 | 0.859 | 0.46 | 0.126 | – | 1.697 | 0.244 | 2.00 | 0.224 |  | 17.894 | 0.535 |
|  |  | Right posterior section (6, 7) | 1.57 | 0.703 | – | 3.489 | 0.272 | 1.02 | 0.311 | – | 3.332 | 0.977 | 0.53 | 0.070 |  | 4.038 | 0.543 |
|  | Lobes | |  |  |  |  |  |  |  |  |  |  |  |  |  |  |  |
|  |  | Right lobes (5, 6, 7, 8) | 1.32 | 0.761 | – | 2.295 | 0.322 | 1.33 | 0.545 | – | 3.262 | 0.529 | 1.47 | 0.250 |  | 8.698 | 0.669 |
|  | Largest diameter of abscess (mm) | | 1.01 | 1.003 | – | 1.024 | 0.013 | 1.01 | 0.998 | – | 1.028 | 0.091 | 1.01 | 0.980 |  | 1.036 | 0.605 |
|  | Number of abscess | |  |  |  |  |  |  |  |  |  |  |  |  |  |  |  |
|  |  | 2 | 0.53 | 0.218 | – | 1.311 | 0.171 | 0.67 | 0.127 |  | 3.511 | 0.632 | 0.00 | 0.000 | – |  | 0.999 |
|  |  | ≤3 | 1.85 | 0.831 | – | 4.101 | 0.132 | 0.71 | 0.234 |  | 2.176 | 0.554 | 0.56 | 0.054 | – | 5.700 | 0.621 |
|  | Culture results of abscess | |  |  |  |  |  |  |  |  |  |  |  |  |  |  |  |
|  |  | K. pneumoniae | 0.92 | 0.475 | – | 1.784 | 0.807 | 1.63 | 0.526 | – | 5.038 | 0.397 | 0.81 | 0.396 | – | 1.657 | 0.564 |
|  |  | Other gram (+) | 1.53 | 0.662 | – | 3.557 | 0.318 | 1.80 | 0.488 | – | 6.635 | 0.377 | 1.31 | 0.525 | – | 3.256 | 0.565 |
|  |  | Mixed | 1.30 | 0.350 | – | 4.858 | 0.692 | 7.20 | 1.009 | – | 51.392 | 0.049 | 0.46 | 0.084 | – | 2.563 | 0.378 |
|  | Blood culture results | |  |  |  |  |  |  |  |  |  |  |  |  |  |  |  |
|  |  | K. pneumoniae | 1.90 | 1.057 | – | 3.432 | 0.032 | 0.71 | 0.297 | – | 1.681 | 0.432 | 1.28 | 0.242 | – | 6.704 | 0.774 |
|  |  | Other gram (+) | 0.90 | 0.324 | – | 2.497 | 0.840 | 0.75 | 0.134 | – | 4.162 | 0.738 | 1.00 | 0.000 | – | 0.000 | 0.999 |
|  |  | Mixed | 2.57 | 0.456 | – | 14.490 | 0.284 | 0.62 | 0.061 | – | 6.332 | 0.688 | 0.00 | 0.000 | – |  | 1.000 |
|  | Recurrence of PLA | | 2.12 | 0.640 | – | 7.479 | 0.212 | 4.03 | 0.908 | – | 17.914 | 0.067 | 1.92 | 0.270 | – | 13.631 | 0.516 |
|  | Location of PE | |  |  |  |  |  |  |  |  |  |  |  |  |  |  |  |
|  |  | Right |  |  |  |  |  | 1.47 | 0.227 | – | 9.556 | 0.684 | 18.00 | 0.585 | – | 553.586 | 0.098 |
|  |  | Bilateral |  |  |  |  |  | 1.00 | 0.444 | – | 2.273 | 0.991 | 20.57 | 2.158 | – | 196.103 | 0.009 |
|  | Presence of PE at diagnosis | |  |  |  |  |  | 1.43 | 0.634 | – | 3.220 | 0.390 | 1.60 | 0.368 | – | 6.959 | 0.531 |
|  | First presence of PE (day) | |  |  |  |  |  | 1.01 | 0.949 | – | 1.073 | 0.780 | 0.99 | 0.892 | – | 1.093 | 0.808 |
|  | Pigtail catheter indwelling periods (day) | |  |  |  |  |  | 1.03 | 0.996 | – | 1.067 | 0.079 | 1.12 | 1.010 | – | 1.251 | 0.032 |

Normal ranges of variables are presented as follows: WBC, 4,000–10,000 /mm^3^; Neutrophil, 38–75% of WBC; CRP, 0–5.0 mg/dL.

OR, odds ratio; HBV, hepatitis B virus; ER, emergency room; ICU, intensive care unit; WBC, white blood cell; CRP, C-reactive protein; PLA, pyogenic liver abscess; PE, pleural effusion.
