# Supplementary material for: Evaluation of clinical risk factors for developing pleural empyema secondary to liver abscess
Source: BMC Gastroenterol. 2019 Dec 16;19:215. doi: 10.1186/s12876-019-1128-4 (PMC6915871; doi:10.1186/s12876-019-1128-4)
Supplement: Supplementary file 2 — Additional file 2: Table S2. Antibiotics treatment regimen. Table S3. Univariate and Multivariate analysis of risk factors for bilateral pleural effusion. Table S4. Classification of combined biliary tract disease according to the Groups. [file 12876_2019_1128_MOESM2_ESM.docx]

| **Variables** | | | **Patient without effusion (N=120)** | **Patient with effusion (N=114)** | | | | | | | **Total (N=234)** | | ***P*-value** |
| --- | --- | --- | --- | --- | --- | --- | --- | --- | --- | --- | --- | --- | --- |
|  |  |  |  | Patients treated medically (N=78) | Patients treated with intervention (N=36) | | | | Total (N=114) | *P*-value |  |  |  |
|  |  |  | Group1 (N=120) | Group2 (N=78) | Group3 (N=26) | Group4 (N=10) | Total (N=36) | *P*-value |  |  |  |  |  |
|  | **Antibiotics regimen** | |  |  |  |  |  | 0.064 |  | 0.859 |  |  | 0.206 |
|  | | 3rd/4th generation cephalosporin  with metronidazole | 106 (88.3) | 65 (83.3) | 23 (11.4) | 7 (70.0) | 30 (83.3) |  | 95 (83.3) |  | 201 (85.9) | |  |
|  |  | Tazobactam/piperacillin | 7 (5.8) | 10 (12.8) | 1 (3.8) | 3 (30.0) | 4 (11.1) |  | 14 (12.3) |  | 21 (9.0) | |  |
|  |  | Others | 7 (5.8) | 1 (3.8) | 2 (7.7) | 0 (0.0) | 2 (5.6) |  | 5 (4.4) |  | 12 (5.1) | |  |
|  | **Changes of regimen** | |  |  |  |  |  | <0.001 |  | 0.734 |  | | <0.001 |
|  |  | None | 79 (65.8) | 35 (44.9) | 18 (69.2) | 1 (10.0) | 19 (52.8) |  | 54 (47.4) |  | 133 (56.8) | |  |
|  |  | Once | 37 (30.8) | 28 (35.9) | 8 (30.8) | 3 (30.0) | 11 (30.6) |  | 39 (34.2) |  | 76 (32.5) | |  |
|  |  | Twice | 4 (3.3) | 15 (19.2) | 0 (0.0) | 6 (60.0) | 6 (16.7) |  | 21 (18.4) |  | 25 (10.7) | |  |

**Additional file 2: Table S2**. Antibiotics treatment regimen.

Data are presented as number (%).

| **Variables** | | | | | **HR for Pleural effusion** | | | | |
| --- | --- | --- | --- | --- | --- | --- | --- | --- | --- |
|  | **Univariate analysis** | | | | **OR** | **95% Confidential Interval** | | | ***P*-value** |
|  |  |  | Comorbidity | |  |  |  |  |  |
|  |  |  |  | Chronic kiney disease | 3.26 | 1.071 | – | 9.896 | 0.037 |
|  |  |  |  | Heart disease | 3.76 | 1.201 | – | 11.789 | 0.023 |
|  |  |  | Combined inflammatory condition | |  |  |  |  |  |
|  |  |  |  | Urinary tract infection | 2.39 | 1.027 | – | 5.560 | 0.043 |
|  |  |  |  | Acute kidney injury | 4.32 | 1.712 | – | 10.925 | 0.002 |
|  |  |  | Metastatic infection | |  |  |  |  |  |
|  |  |  |  | Sepsis | 2.70 | 1.184 | – | 6.166 | 0.018 |
|  |  |  | Asctes | | 3.99 | 1.936 | – | 8.233 | <0.001 |
|  |  |  | Admission to ICU | | 7.41 | 3.452 | – | 15.894 | <0.001 |
|  |  |  | ICU care during treatment periods | | 5.68 | 2.813 | – | 11.464 | <0.001 |
|  |  |  | Use of Mechanical ventilation | | 8.61 | 1.526 | – | 48.512 | 0.015 |
|  |  |  | Location of largest abscess | |  |  |  |  |  |
|  |  |  | Association with diaphragm | |  |  |  |  |  |
|  |  |  |  | Near Left diaphragm (2, 4) | 2.63 | 1.079 | – | 6.389 | 0.033 |
|  |  |  |  | Near Right. diaphragm (7, 8) | 2.00 | 0.886 | – | 4.516 | 0.095 |
|  |  |  | Culture results of abscess | |  |  |  |  |  |
|  |  |  |  | K.pneumoniae | 2.04 | 0.791 | – | 5.257 | 0.140 |
|  |  |  |  | Other gram (+) | 1.70 | 0.536 | – | 5.373 | 0.368 |
|  |  |  |  | Mixed | 2.33 | 0.489 | – | 11.123 | 0.288 |
|  |  |  | Presence of PE at diagnosis | | 6.39 | 3.069 | – | 13.283 | <0.001 |
|  | **Multivariate analysis** | | | | **OR** | **95% Confidential Interval** | | | ***P*-value** |
|  |  |  | Acute kidney injury | | 3.83 | 1.227 | – | 11.958 | 0.021 |
|  |  |  | Asctes | | 2.46 | 1.068 | – | 5.653 | <0.001 |
|  |  |  | Presence of PE at diagnosis | | 5.59 | 2.306 | – | 13.535 | <0.001 |

**Table S3** Univariate and Multivariate analysis of risk factors for bilateral pleural effusion.

OR, odds ratio; ICU, intensive care unit; PE, pleural effusion.

| **Variables** | | | **Patient without effusion (N=120)** | **Patient with effusion (N=114)** | | | | | | | **Total (N=234)** | ***P*-value** |
| --- | --- | --- | --- | --- | --- | --- | --- | --- | --- | --- | --- | --- |
|  |  |  |  | Patients treated medically (N=78) | Patients treated with intervention (N=36) | | | | Total (N=114) | *P*-value |  |  |
|  |  |  | Group1 (N=120) | Group2 (N=78) | Group3 (N=26) | Group4 (N=10) | Total (N=36) | *P*-value |  |  |  |  |
|  | Combined bilary tract disease | |  |  |  |  |  | 0.077 |  | 0.311 |  | 0.023 |
|  |  | Cholecystitis | 20 (16.7) | 18 (12.1) | 2 (7.7) | 3 (30.0) | 5 (13.8) |  | 23 (20.2) |  | 43 (18.4) |  |
|  |  | Cholantitis | 3 (2.5) | 4 (5.1) | 2 (7.7) | 0 (0.0) | 2 (5.6) |  | 6 (5.3) |  | 9 (3.8) |  |
|  |  | Choledocholithiasis | 0 (0.0) | 2 (2.6) | 2 (7.7) | 2 (20.0) | 4 (11.1) |  | 6 (5.3) |  | 6 (2.6) |  |
|  |  | others | 0 (0.0) | 1 (1.3) | 0 (0.0) | 1 (10.0) | 1 (2.8) |  | 2 (1.8) |  | 2 (0.9) |  |
|  |  | Total | 23 (19.2) | 25 (32.1) | 6 (23.1) | 6 (60.0) | 12 (33.3) |  | 36 (25.2) |  | 60 (25.6) |  |
|  | Previous History | |  |  |  |  |  | 0.452 |  | 0.137 |  | 0.114 |
|  |  | Cholecystitis | 20 (16.7) | 13 (16.6) | 2 (7.7) | 2 (20.0) | 4 (11.1) |  | 17 (14.9) |  | 37 (15.9) |  |
|  |  | Cholantitis | 1 (0.8) | 2 (2.6) | 2 (7.7) | 0 (0.0) | 2 (5.6) |  | 4 (3.5) |  | 5 (2.1) |  |
|  |  | Choledocholithiasis | 0 (0.0) | 1 (1.3) | 0 (0.0) | 1 (10.0) | 4 (11.1) |  | 5 (4.4) |  | 5 (2.1) |  |
|  |  | others | 0 (0.0) | 0 (0.0) | 2 (7.7) | 1 (10.0) | 1 (2.8) |  | 1 (0.9) |  | 1 (0.4) |  |
|  |  | Total | 21 (17.5) | 16 (20.5) | 6 (23.1) | 5 (50.0) | 11 (30.6) |  | 27 (23.7) |  | 48 (20.5) |  |

**Table S 4** Classification of combined biliary tract disease according to the Groups

Data are presented as number (%).
